# Supplementary material for: ESKAPE Gram-negative bacteria escape culture-based detection upon desiccation on abiotic surfaces
Source: Microbiol Spectr. 2026 Feb 4;14(3):e03357-25. doi: 10.1128/spectrum.03357-25 (PMC12955428; doi:10.1128/spectrum.03357-25)
Supplement: Supplemental material — Tables S1 to S4, Fig. S1 to S7, and supplemental methods. [file spectrum.03357-25-s0001.pdf]

Supplemental Material

ESKAPE Gram-negative bacteria escape culture-based detection upon desiccation on abiotic surfaces

Daniela Visaggio[a,b,c*], Massimiliano Lucidi[a,b*], Cinzia Spagnoli[a], Ilaria Ciccone[a], Francesco Imperi[a,b,c], Paolo Visca[a,b,c]

[a] Department of Science, Roma Tre University, Rome, Italy

[b]NBFC, National Biodiversity Future Center, Palermo, Italy

[c]Santa Lucia Foundation IRCCS, Rome, Italy

*Equally contributed

✉ Address for correspondence: D. Visaggio, Department of Science, Roma Tre University, Viale G. Marconi 446, 00146 Rome, Italy (daniela.visaggio@uniroma3.it); P. Visca, Department of Science, Roma Tre University, Viale G. Marconi 446, 00146 Rome, Italy (paolo.visca@uniroma3.it).

Table of contents

Table S1	ESKAPE species capable of entering the VBNC state upon exposure to different types of stress	ii
Table S2	Bacterial strains used in this study	iii
Table S3	Substrate materials used in desiccation assays	iv
Table S4	List of the swabs and chemical composition of the suspension media used in this study	v
Figure S1	Resistance to desiccation and entrance into the VBNC state after desiccation of ESKAPE bacteria	vi
Figure S2	Growth of ESKAPE bacteria in RB supplemented or not with LB	viii
Figure S3	Statistical analysis of bacterial cultivability after desiccation on the tested surfaces	x
Figure S4	CLSM imaging and membrane integrity analysis of LIVE/DEAD-stained ESKAPE bacteria before and after desiccation, and after resuscitation	xi
Figure S5	Comparison of the resuscitation procedure with standard techniques used for environmental contamination control	xiii
Figure S6	Growth profile of ESKAPE bacteria in different transport buffers	xv
Figure S7	Resuscitation assay in RB and Amies solution	xvi
Macro Script for CLSM image processing	Script for quantification of bacterial membrane integrity	xvii
Protocol	Surface sampling for the detection of Gram-negative bacteria in VBNC state	xviii
References		xx

Table S1. ESKAPE species capable of entering the VBNC state upon exposure to different types of stress

Bacterial species	Stress	Reference
Enterococcus spp.	Chlorination	Jiang et al., 2023
	Nutrient limitation	Zhang et al., 2024; Gin et al., 2013; Lleo et al., 2007; Lleo et al., 2005; Signoretto et al., 2005; Lleo et al., 2003; Heim et al., 2002; Lleo et al., 2001
Staphylococcus aureus	Chitooligosaccharides	Li et al., 2024
	High pressure	Yang et al., 2023
	Citral and cinnamaldehyde	Cheng et al., 2023
	Low temperature (Frozen)	Yan et al., 2021
	Nutrient limitation	Li et al., 2020
	Non-thermal plasma	Liao et al., 2020
	Citric acid	Bai et al., 2019
	Antibiotics	Pasquaroli et al., 2014; Pasquaroli et al., 2013
Klebsiella pneumoniae	High salinity and micro-aerophilic conditions	Zhao et al., 2024
	Formic acid and organic acids (food preservatives)	Yadav et al., 2022
	Oxidative stress (H_2O_2)	Alveraz-Daza et al., 2021
Acinetobacter baumannii	Desiccation	Konig et al., 2023; Lucidi et al., 2025
	Formic acid and organic acids	Yadav et al., 2022
Pseudomonas aeruginosa	Low oxygen	Kvich et al., 2019
	Antibiotics	Mangiaterra et al., 2023; Mangiaterra et al., 2020
	Chlorine	Qi et al., 2023; Qi et al., 2022; Chiang et al., 2022; Wang et al., 2022
	UV	Wang et al., 2021, Zhang et al., 2015
	Copper toxicity	Dopp et al., 2017; Bedard et al., 2014

Table S2. Bacterial strains used in this study

Bacterial species	Strain	Country	Year	Source	Resistance	Reference
Enterococcus faecalis	ATCC 29212	ns	ns	urine	ns	American Type Culture Collection
E. faecalis	ATCC 700802	USA	1987	blood	VAN (*vanB*), GEN	Sahm et al., 1989
Enterococcus faecium	ATCC 19434^T	ns	ns	unknown	ns	Schleifer and Kilpper-Balz, 1984
E. faecium	BM4147	ns	ns	human clinical	VAN (*vanA*)	Bugg et al., 1991
S. aureus	ATCC 43300	USA	ns	human clinical	MRSA	American Type Culture Collection
S. aureus	UD95	Italy	2011	human clinical	MDR	Hijazi et al., 2018
K. pneumoniae	ATCC 27736	ns	ns	human clinical	ns	American Type Culture Collection
K. pneumoniae	Kp3	Italy	2011	blood	MDR	Villa et al., 2014
A. baumannii	ATCC 17978	France	1951	blood	SXT	Smith et al., 2007
A. baumannii	Ab_2	Italy	2020	blood	MDR	This work
P. aeruginosa	ATCC 15692 (PAO1)	ns	ns	infected wound	ns	American Type Culture Collection
P. aeruginosa	SP-13	Italy	ns	blood	MDR	Bonchi et al., 2015
Enterobacter aerogenes	ATCC 13048^T	USA	ns	sputum	ns	Bascomb et al., 1971
E. aerogenes	61-4945	Italy	2016	blood	-	This work
Enterobacter cloacae	ATCC 13047^T	USA	ns	spinal fluid	susceptible	Hormaeche and Edwards, 1960
E. cloacae	65-6880	Italy	2016	blood	-	This work

Abbreviations: GEN, gentamicin; MDR, multidrug-resistant; MRSA, methicillin-resistant *Staphylococcus aureus*; SXT, trimethoprim-sulfamethoxazole; VAN, vancomycin; ns, not specified.^{T} type strain.

Table S3. Substrate materials used in desiccation assays

Materials	Abbreviation	Some examples of hospital devices
Glass	GL	IV bottles, ampoules, vials, blood collection tubes
Polyvinyl chloride	PVC	Primary IV tubes and bags, blood bags, oxygen masks, endotracheal tubes, drainage bags and tubes, respiratory tubing, infusion sets, dialysis bags and tubing
Polypropylene	PP	Disposable scrubs, suture materials, and medical packaging
Polyester	PL	Surgical masks, vascular grafts, surgical mesh, implantable textiles, medical tapes and bandages, pillow and mattress covers, surgical drapes, and sterilization wraps
Polystyrene	PS	Tubes, sample containers, trays and instrument holders, and disposable syringes
Polyethylene	PE	Urinal bottles, IV tubing, syringe components, drains, oxygen masks
Silicone	SL	Catheters, endotracheal tubes, ostomy bags, wound dressings, infusion devices
Cotton	CT	Lab coats, cotton balls, cotton swabs, cotton gauze pads, cotton bandages
Titanium	Ti	Prosthetics, orthopedic, dental, cardiovascular, craniofacial and maxillofacial, neurosurgical implants and surgical instruments

Abbreviations: IV, intravenous

Table S4. List of the swabs and chemical composition of the suspension media used in this study

Swab	Abbreviation	Material	Buffer composition (g/L)	Provider
FLOQSwabs	RB	Nylon	Na_2HPO_4 (6.80)<br>KH_2PO_4 (3.00)<br>NaCl (0.50)<br>NH_4Cl (1.00)<br>$CaCl_2$ (0.02)<br>$MgSO_4$ (0.12)	Swab: Copan<br>Salt powder: Merk
ESC Swab D/E Neutralizing Broth[a]	NB	Rayon	Enzymatic digest of casein (5.00)<br>Yeast extract (2.50)<br>Dextrose (10.00)<br>Sodium thioglycolate (1.00)<br>$Na_2S_2O_3$ (6.00)<br>$NaHSO_3$ (2.50)<br>Lecithin; (7.00)<br>Polysorbate 80; (5.00)<br>Bromocresol purple (0.02)	Liofilchem
ESC Swab Maximum Recovery Diluent[b]	MRD	Rayon	Enzymatic digest of casein (1.00)<br>NaCl (8.50)	Liofilchem
ESC Swab Neutralizing Rinse Solutions[c]	NRS	Rayon	Casein peptone (1.00)<br>Histidine (1.00)<br>Lecithin (2.00)<br>Tween 80 (30.00)<br>$Na_2S_2O_3$ (5.00)<br>NaCl (8.50)	Liofilchem
LMS swab Amies[d]	Amies	Polyurethane foam	Na_2HPO_4 (1.15)<br>KH_2PO_4 (0.20)<br>NaCl (3.00)<br>KCl (0.20)<br>$CaCl_2$ (0.10)<br>$MgCl_2$ (0.10)<br>$C_2H_3NaO_2S$ (1.00)	Heinz Herenz Medizinalbedarf GmbH

Abbreviations: ESC, easy surface checking; LMS, transport system with liquid medium.

[a] the neutralizing agents in the medium inactivate residual antimicrobials. Sodium thiosulfate, sodium bisulfite, lecithin, and polysorbate 80 neutralize a broad spectrum of antiseptics and disinfectants, including quaternary ammonium compounds, phenolics, iodine, chlorine preparations, mercurials, formaldehyde, and glutaraldehyde.

[b] the buffer contains an enzymatic digest of casein at low concentration and physiological saline, allowing the recovery of even stressed or injured cells.

[c] the buffer contains the following neutralizers of disinfectants: L-histidine (inactivates aldehydes), lecithin (neutralizes the quaternary ammonium compounds), polysorbate 80 (inactivates phenolic compounds and mercurial derivatives), and sodium thiosulfate (neutralizes halogen compounds).

[d] transport medium for the sampling and preservation of aerobic and anaerobic bacteria at room or refrigerated temperature.

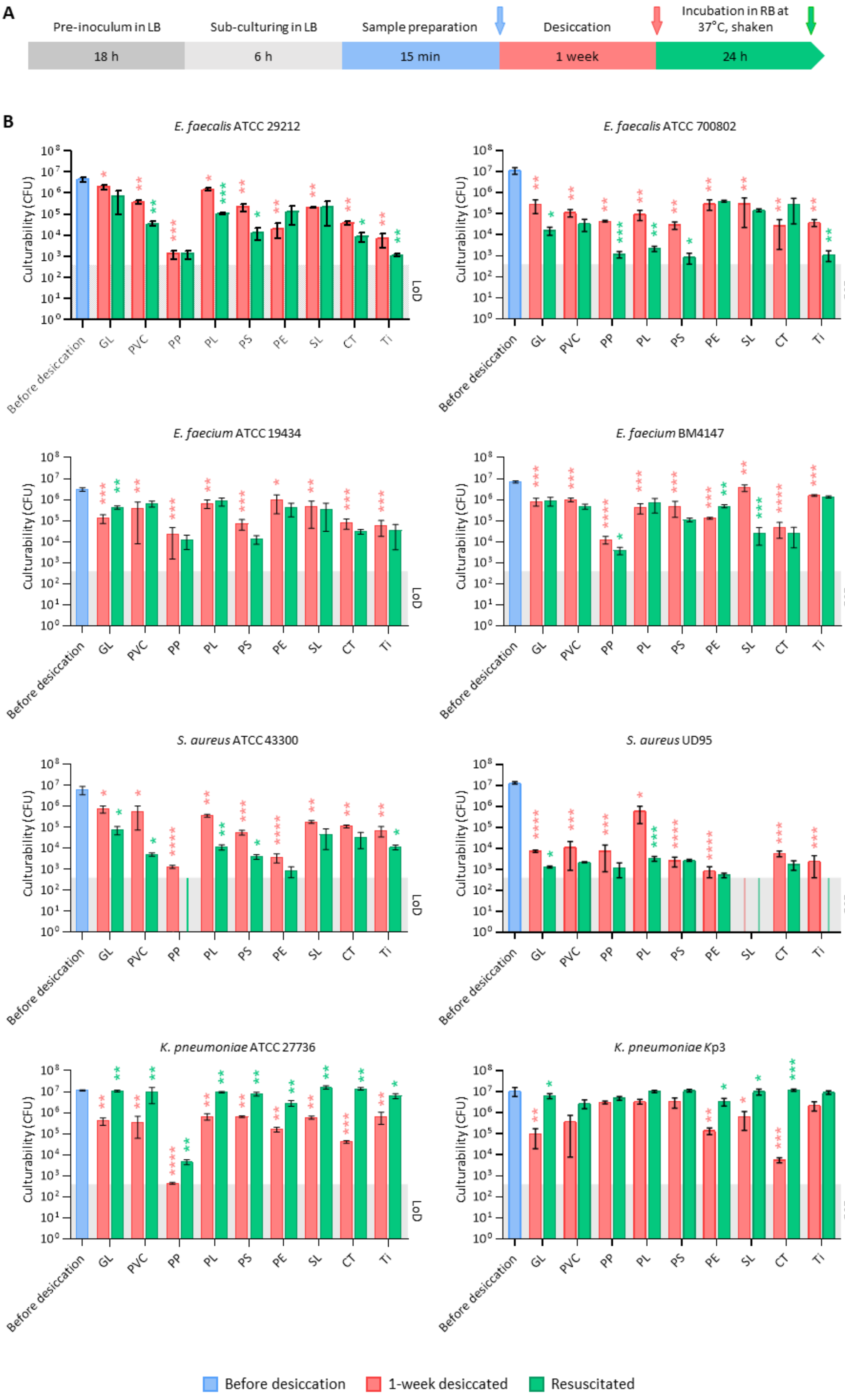
A
Pre-inoculum in LB
Sub-culturing in LB
Sample preparation
Desiccation
Incubation in RB at 37°C, shaken
18 h
6 h
15 min
1 week
24 h
B
E. faecalis ATCC 29212
E. faecalis ATCC 700802
E. faecium ATCC 19434
E. faecium BM4147
S. aureus ATCC 43300
S. aureus UD95
K. pneumoniae ATCC 27736
K. pneumoniae Kp3
Culturability (CFU)
LoD
Before desiccation
GL
PVC
PP
PL
PS
PE
SL
CT
Ti
Before desiccation
1-week desiccated
Resuscitated

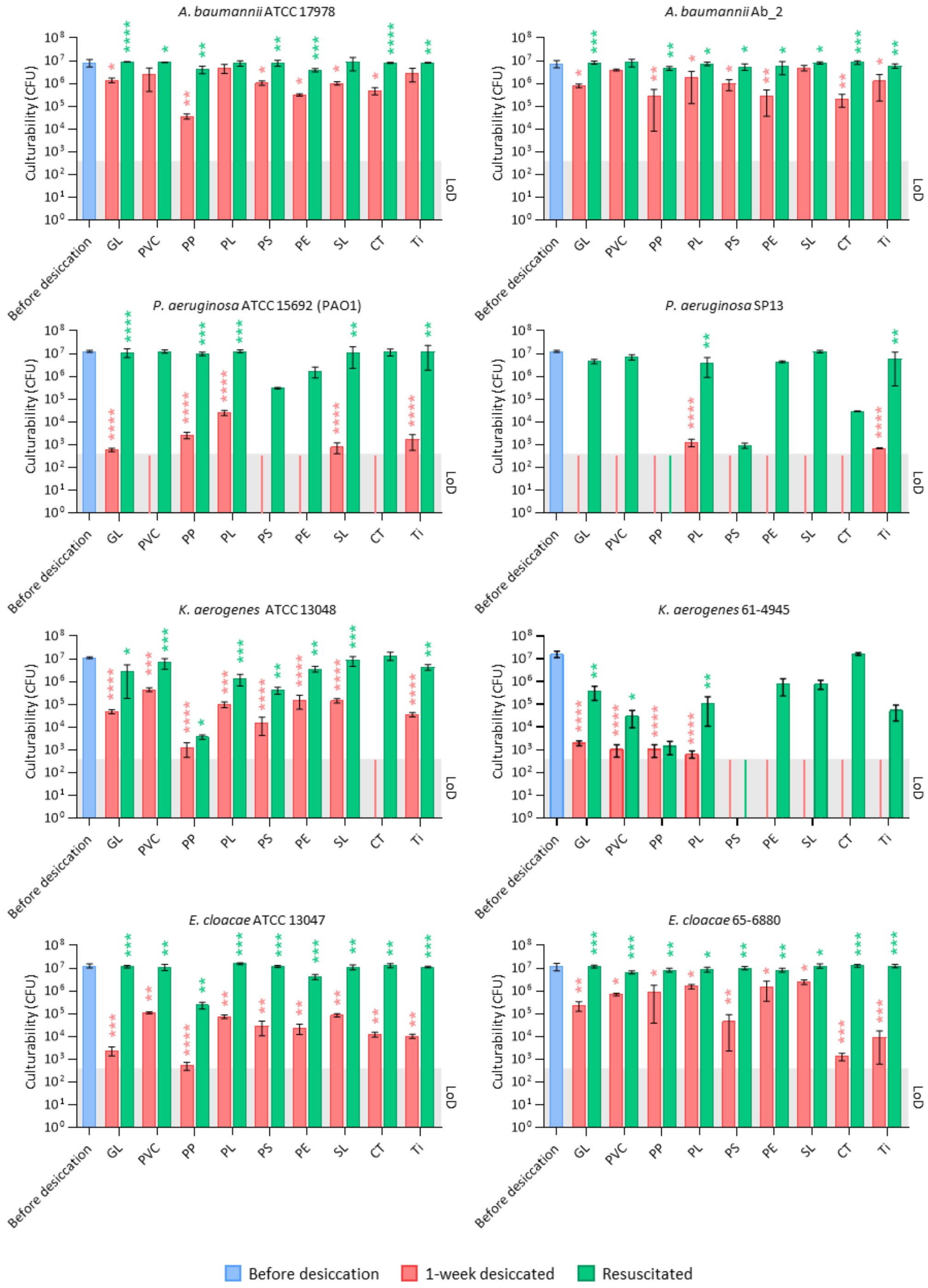

FIG S1 Resistance to desiccation and entrance into VBNC state after desiccation of ESKAPE bacteria. (**A**) Experimental timeline of the desiccation resistance assay. Colored arrows indicate the sampling times for CFU counts; cyan, after sample preparation in double-distilled water (before desiccation); red, after one-week desiccation; green, after resuscitation. (**B**) Bacterial strains were air-dried for one week on glass (GL), polyvinylchloride (PVC), polypropylene (PP), polyester (PL), polystyrene (PS), polyethylene (PE), silicon (SL), cotton (CT), and titanium (Ti). After desiccation, cells were suspended in RB and incubated at 37°C for 24 h with shaking. Cultivability was determined before desiccation, after one-week desiccation, and after resuscitation. The grey area indicates the limit of detection (LoD), corresponding to 4×10^2 CFU, with red and green segments representing CFU values below the LoD after desiccation and resuscitation, respectively. Data are the mean ± SD (error bars) of three independent experiments. Red asterisks indicate statistically significant differences between samples before and after desiccation for a given surface. Green asterisks indicate statistically significant differences between samples after desiccation on a given surface and after resuscitation. Statistically significant differences (*$P < 0.05$; **$P < 0.01$; ***$P < 0.001$; ****$P < 0.0001$) were determined using the unpaired *t*-test.

E. faecalis ATCC 29212

	GL	PVC	PP	PL	PS	PE	SL	CT
PVC	0.0001							
PP	0.0001	0.4128						
PL	0.3256	0.0001	0.0001					
PS	0.0001	0.9893	0.9033	0.0001				
PE	0.0001	0.4822	0.9999	0.0001	0.9406			
SL	0.0001	0.9807	0.9319	0.0001	0.9999	0.9610		
CT	0.0001	0.5405	0.9999	0.0001	0.9625	0.9999	0.9770	
Ti	0.0001	0.4328	0.9999	0.0001	0.9155	0.9999	0.9416	0.9999

E. faecalis ATCC 700802

	GL	PVC	PP	PL	PS	PE	SL	CT
PVC	0.7496							
PP	0.3617	0.9986						
PL	0.6458	0.9999	0.9998					
PS	0.2939	0.9947	0.9999	0.9990				
PE	0.9999	0.6241	0.2636	0.5166	0.2097			
SL	0.9999	0.6825	0.3056	0.5751	0.2454	0.9999		
CT	0.2845	0.9937	0.9999	0.9988	0.9999	0.2024	0.2372	
Ti	0.3362	0.9976	0.9999	0.9997	0.9999	0.2431	0.2828	0.9999

E. faecium ATCC 19434

	GL	PVC	PP	PL	PS	PE	SL	CT
PVC	0.9841							
PP	0.9999	0.8960						
PL	0.5248	0.9684	0.3128					
PS	0.9999	0.9503	0.9999	0.4048				
PE	0.1127	0.5008	0.0534	0.9796	0.0761			
SL	0.9423	0.9999	0.7868	0.9934	0.8729	0.6475		
CT	0.9999	0.9546	0.9999	0.4154	0.9999	0.0790	0.8807	
Ti	0.9999	0.9383	0.9999	0.3791	0.9999	0.0694	0.8522	0.9999

E. faecium BM4147

	GL	PVC	PP	PL	PS	PE	SL	CT
PVC	0.9999							
PP	0.5117	0.2777						
PL	0.9759	0.8444	0.9755					
PS	0.9922	0.9123	0.9421	0.9999				
PE	0.6898	0.4228	0.9999	0.9971	0.9887			
SL	0.0001	0.0001	0.0001	0.0001	0.0001	0.0001		
CT	0.5664	0.3184	0.9999	0.9860	0.9622	0.9999	0.0001	
Ti	0.6131	0.8588	0.0186	0.1393	0.1878	0.0338	0.0008	0.0224

S. aureus ATCC 43300

	GL	PVC	PP	PL	PS	PE	SL	CT
PVC	0.8954							
PP	0.0025	0.0409						
PL	0.2351	0.9263	0.3743					
PS	0.0053	0.0828	0.9999	0.5825				
PE	0.0025	0.0422	0.9999	0.3824	0.9999			
SL	0.0275	0.3232	0.9530	0.9552	0.9952	0.9563		
CT	0.0108	0.1550	0.9979	0.7831	0.9999	0.9982	0.9999	
Ti	0.0064	0.0991	0.9999	0.6406	0.9999	0.9999	0.9980	0.9999

S. aureus UD95

	GL	PVC	PP	PL	PS	PE	SL	CT
PVC	0.9999							
PP	0.9999	0.9999						
PL	0.0033	0.0035	0.0033					
PS	0.9999	0.9999	0.9999	0.0030				
PE	0.9999	0.9999	0.9999	0.0029	0.9999			
SL	0.9999	0.9999	0.9999	0.0029	0.9999	0.9999		
CT	0.9999	0.9999	0.9999	0.0032	0.9999	0.9999	0.9999	
Ti	0.9999	0.9999	0.9999	0.0030	0.9999	0.9999	0.9999	0.9999

K. pneumoniae ATCC 27736

	GL	PVC	PP	PL	PS	PE	SL	CT
PVC	0.9999							
PP	0.2557	0.3764						
PL	0.7277	0.5710	0.0098					
PS	0.7956	0.6467	0.0126	0.9999				
PE	0.7988	0.9107	0.9808	0.0727	0.0920			
SL	0.9522	0.8685	0.0298	0.9997	0.9999	0.1936		
CT	0.3727	0.5195	0.9999	0.0165	0.0214	0.9971	0.0495	
Ti	0.7625	0.6089	0.0111	0.9999	0.9999	0.0818	0.9999	0.0188

K. pneumoniae Kp3

	GL	PVC	PP	PL	PS	PE	SL	CT
PVC	0.9999							
PP	0.0039	0.0100						
PL	0.0013	0.0033	0.9997					
PS	0.0016	0.0042	0.9999	0.9999				
PE	0.9999	0.9999	0.0045	0.0015	0.0019			
SL	0.9924	0.9999	0.0230	0.0077	0.0097	0.9955		
CT	0.9999	0.9994	0.0029	0.0010	0.0012	0.9999	0.9801	
Ti	0.0602	0.1403	0.9027	0.6330	0.6982	0.0686	0.2750	0.0456

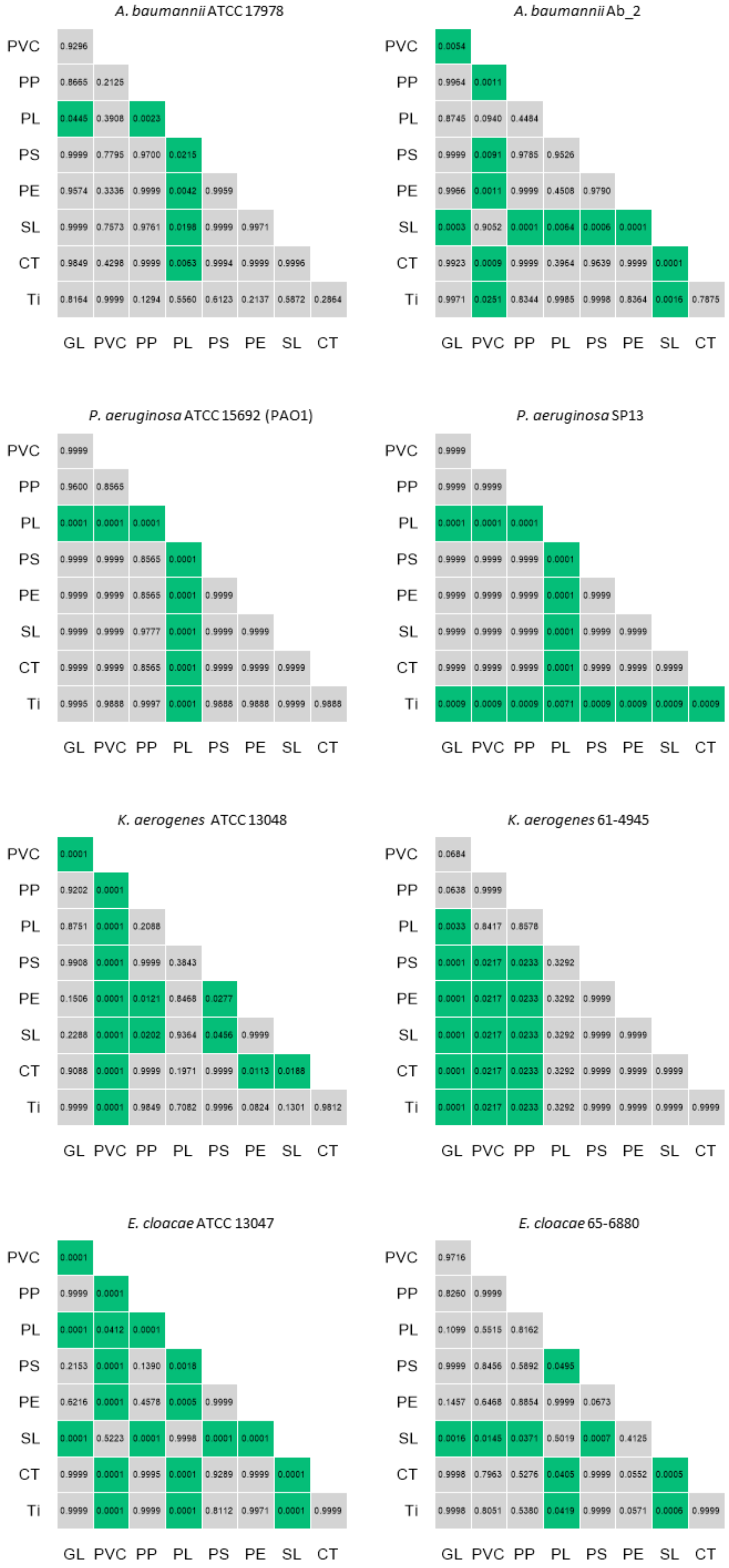

FIG S2. Statistical significance of the observed differences in bacterial cultivability after desiccation on different surfaces. One-way ANOVA followed by Tukey's HSD test was performed to compare the loss of cultivability after desiccation on the different surfaces, as illustrated by the red bars in Fig. S1. Numerical values of the post-hoc statistical comparisons are displayed; P values < 0.05 are highlighted in green, indicating statistically significant differences between surfaces in CFU counts of individual bacterial strains after desiccation.

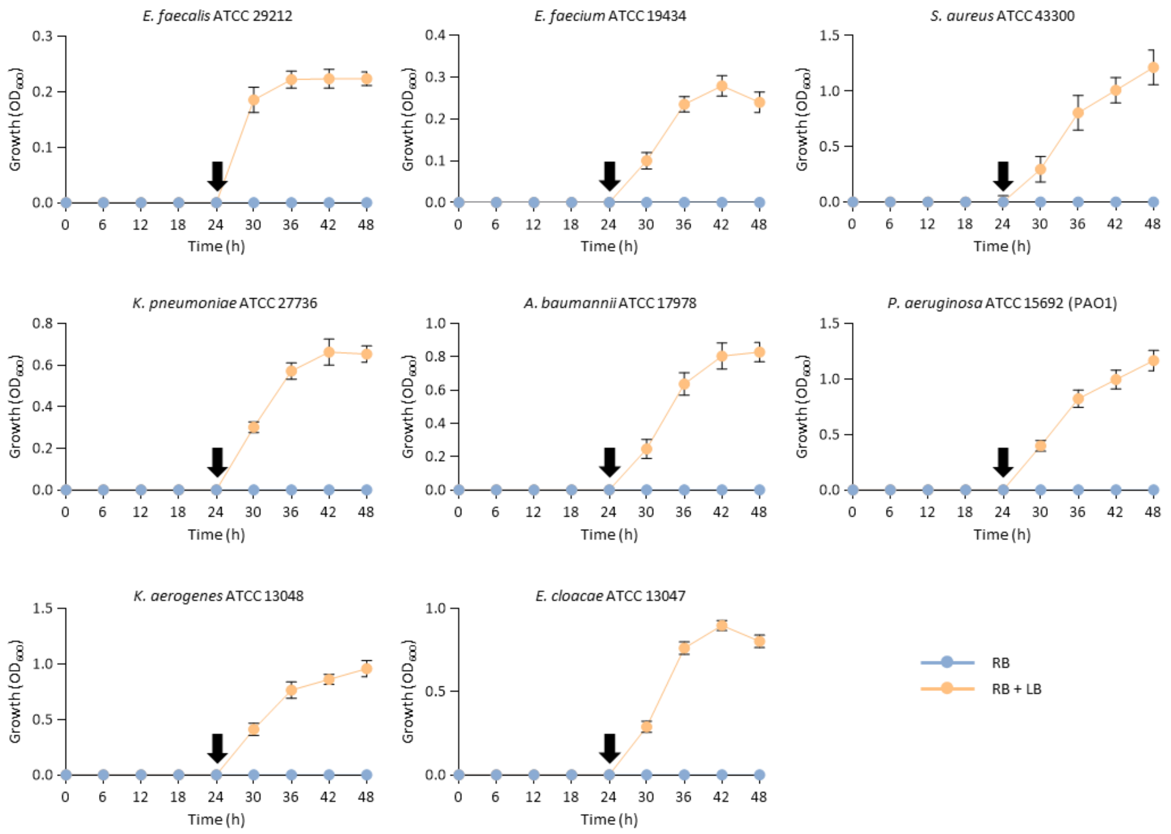

FIG S3 Growth of ESKAPE bacteria in RB supplemented or not with LB. The reference strains of ESKAPE bacteria were incubated at 37°C in RB (cyan) or in 2×RB supplemented, after 24 h, with an equal volume of 2×LB (orange). Black arrows indicate the time point at which 2×LB was added to 2×RB cultures. OD_{600} was periodically measured for up to 48 h. Data are the mean ± SD (error bars) of three independent experiments.

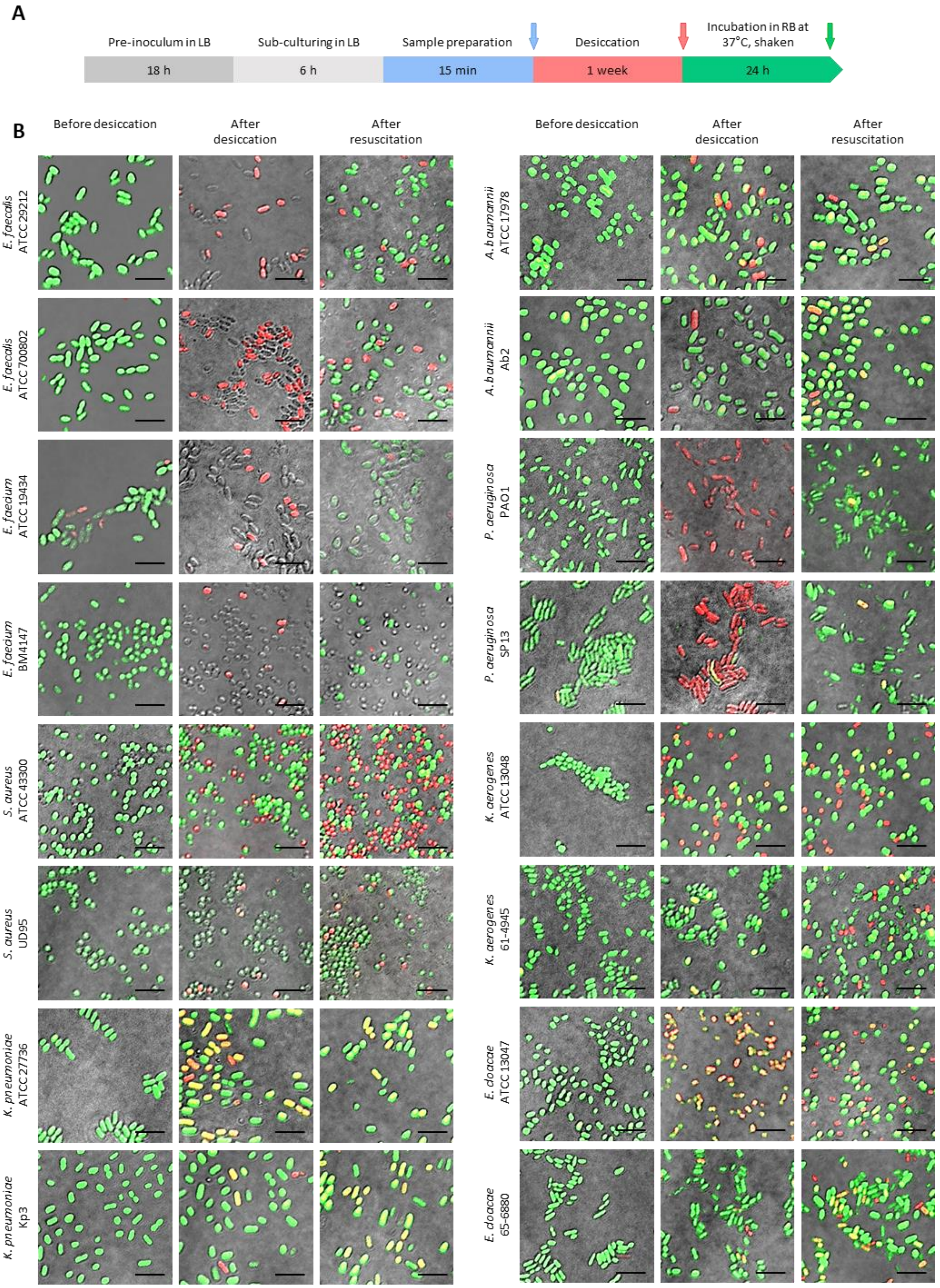
A
Pre-inoculum in LB
Sub-culturing in LB
Sample preparation
Desiccation
Incubation in RB at 37°C, shaken
18 h
6 h
15 min
1 week
24 h
B
Before desiccation
After desiccation
After resuscitation
E. faecalis ATCC 29212
E. faecalis ATCC 700802
E. faecium ATCC 19434
E. faecium BM4147
S. aureus ATCC 43300
S. aureus UD95
K. pneumoniae ATCC 27736
K. pneumoniae Kp3
A. baumannii ATCC 17978
A. baumannii Ab2
P. aeruginosa PAO1
P. aeruginosa SP13
K. aerogenes ATCC 13048
K. aerogenes 61-4945
E. cloacae ATCC 13047
E. cloacae 65-6880

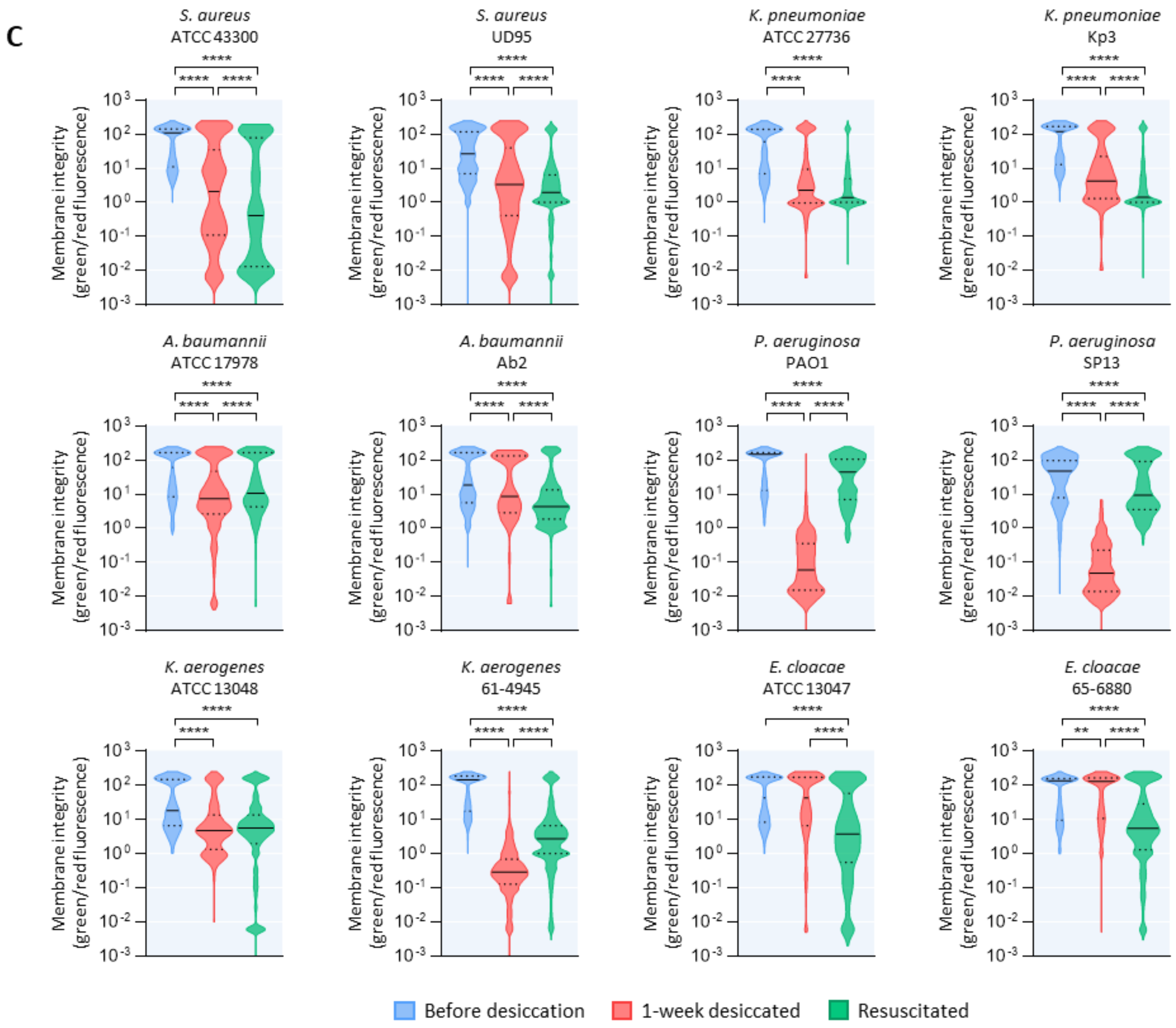

FIG S4 CLSM imaging and membrane integrity analysis of LIVE/DEAD-stained ESKAPE bacteria before and after desiccation, and after resuscitation. (**A**) Schematic of the experimental timeline. Colored arrows indicate the sampling times for LIVE/DEAD staining; cyan, after sample preparation in double-distilled water (before desiccation); red, after one week of desiccation on glass; green, after resuscitation. (**B**) Before desiccation, after desiccation, and after resuscitation, the bacterial suspensions were stained with 10 µM SYTO 9 (green fluorescence) to label intact cells and 60 µM PI (red fluorescence) to mark membrane-damaged cells. Stained bacterial cells were then examined using CLSM. Representative images display the merged channels of differential interference contrast (DIC), SYTO 9, and PI. Scale bar: 5 µm. (**C**) From five CLSM images *per* sample, the green-to-red fluorescence ratio was calculated for each cell to quantitatively assess membrane integrity (n >350 cells *per* sample). The resulting violin plots display the distribution of membrane integrity before desiccation (cyan), after desiccation (red), and following resuscitation (green). The median (filled lines) and interquartile ranges (dashed lines) are shown. Statistical significance was determined by the unpaired t-test (**$P < 0.01$; ****$P < 0.0001$).

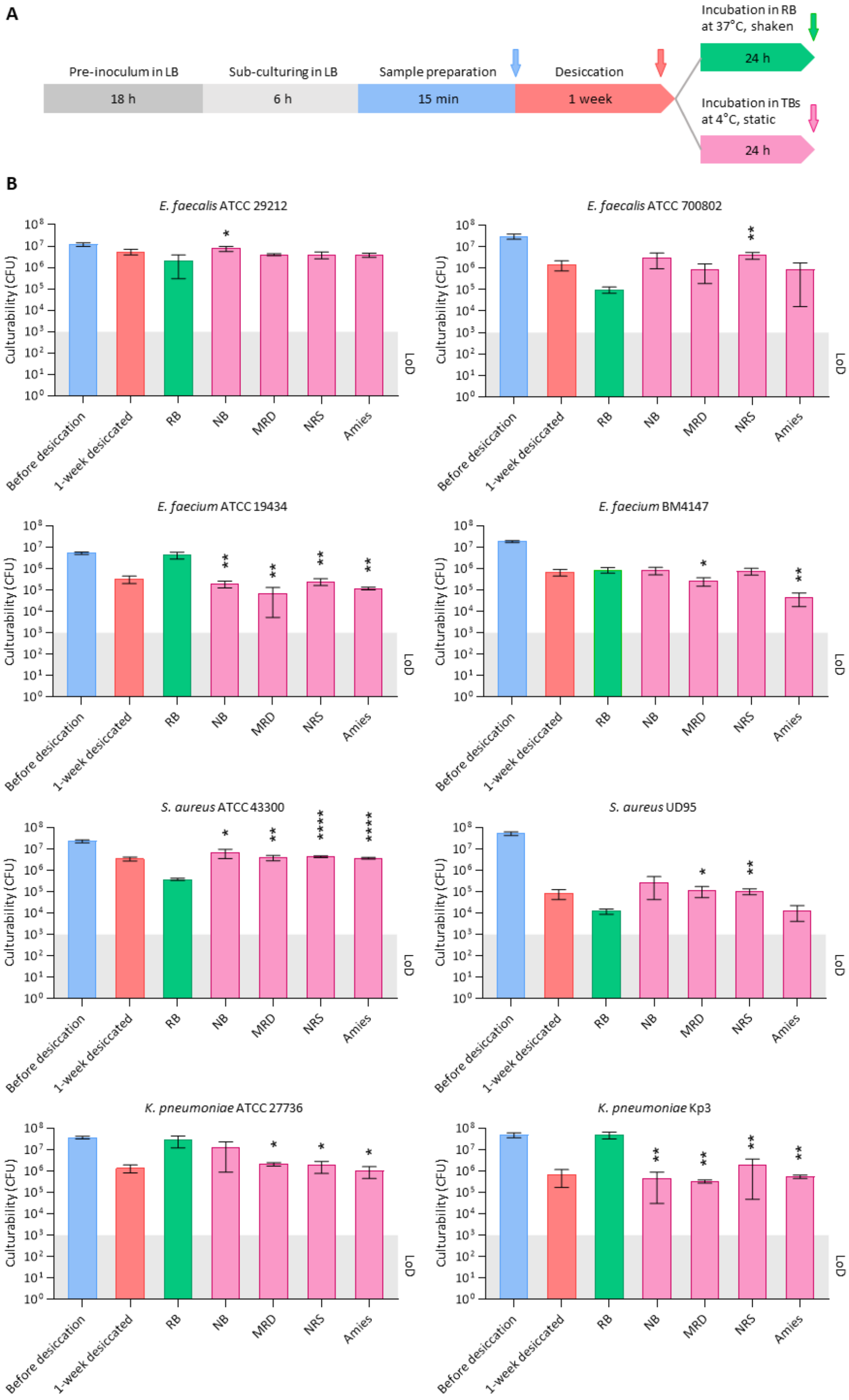
A
Pre-inoculum in LB
18 h
Sub-culturing in LB
6 h
Sample preparation
15 min
Desiccation
1 week
Incubation in RB at 37°C, shaken
24 h
Incubation in TBs at 4°C, static
24 h
B
E. faecalis ATCC 29212
E. faecalis ATCC 700802
E. faecium ATCC 19434
E. faecium BM4147
S. aureus ATCC 43300
S. aureus UD95
K. pneumoniae ATCC 27736
K. pneumoniae Kp3
Culturability (CFU)
LoD
Before desiccation
1-week desiccated
RB
NB
MRD
NRS
Amies

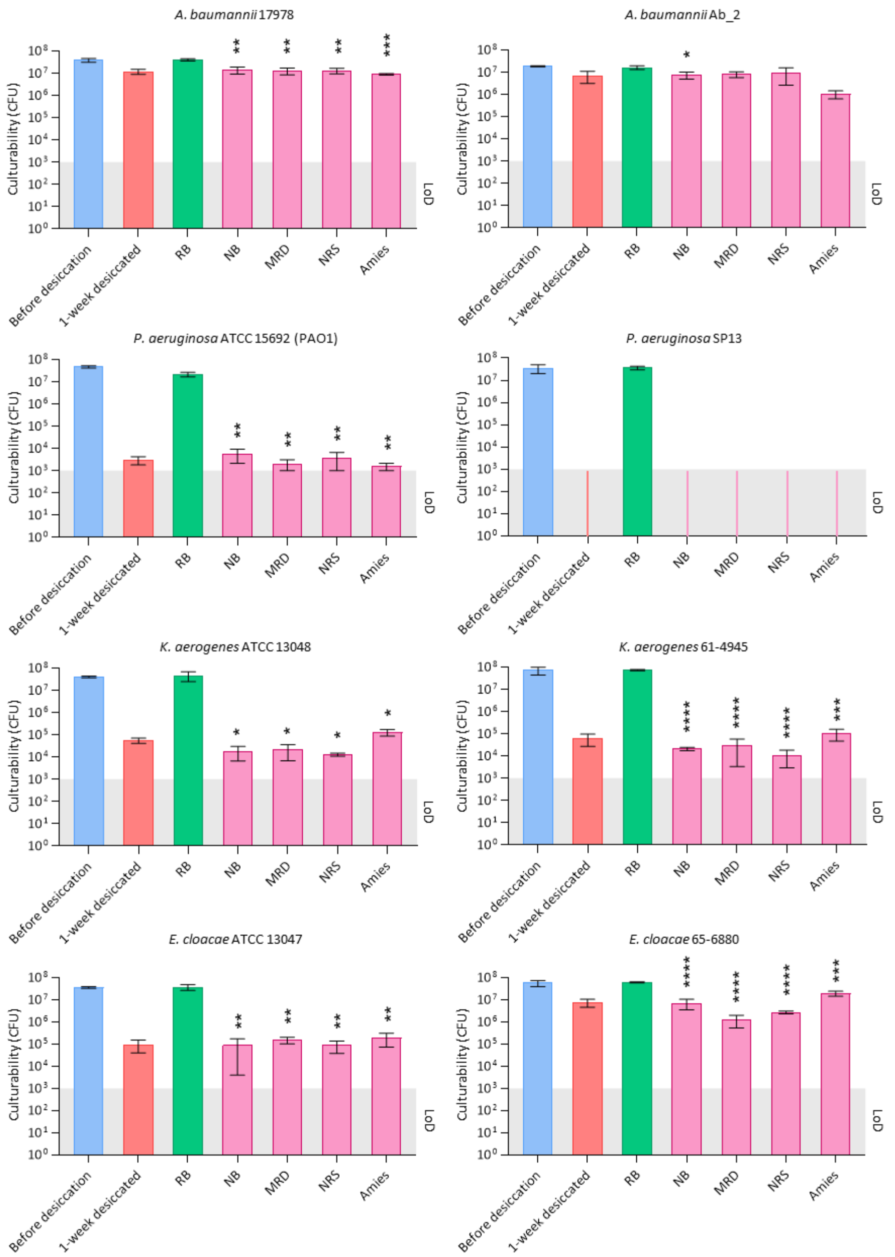

FIG S5. Comparison of the resuscitation procedure with the standard techniques used for environmental contamination control. (**A**) Experimental timeline. Bacterial strains were air-dried for one week on glass. After desiccation, cells were collected using a swab soaked in RB or different commercial swabs used for environmental contamination control. The swabs in RB were incubated at 37°C for 24 h with shaking (resuscitation) while commercial swabs were placed in their respective transport buffer and incubated at 4°C for 24 h in static conditions. Colored arrows indicate the sampling times for CFU counts: cyan, after sample preparation in double-distilled water (before desiccation); red, after one week of desiccation; green, after resuscitation in RB, and pink after incubation of commercial swabs at 4°C for 24 h in static. **(B)** Cultivability of cells after sample preparation in double-distilled water, after one-week desiccation, after resuscitation in RB, and after incubation at 4°C in transport buffer. Transport buffers: Neutralizing Broth (NB), Maximum Recovery Diluent (MRD), Neutralizing Rinse Solution (NRS), and Amies. The grey area indicates the limit of detection (LoD), corresponding to $1{\times}10^3$ CFU, with red and yellow segments representing CFU values below the LoD after desiccation and resuscitation, respectively. Data are the mean ± SD (error bars) of three independent experiments. Asterisks indicate statistically significant differences between CFU counts after resuscitation in RB and after the incubation at 4°C in the TBs. Statistically significant differences (*$P < 0.05$; **$P < 0.01$; ***$P < 0.001$; ****$P < 0.0001$) were determined using the unpaired *t*-test.

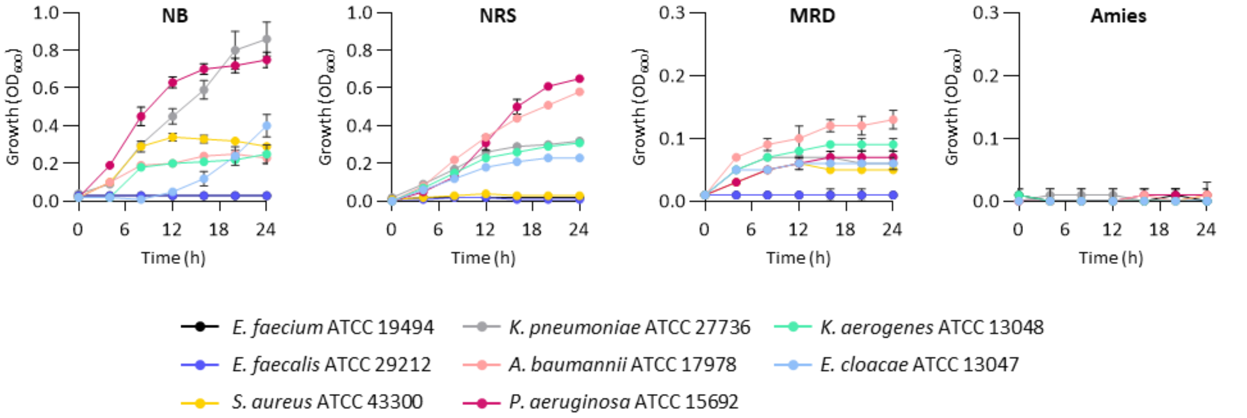

FIG S6. Growth profile of ESKAPE bacteria in different transport buffers. The reference strains of ESKAPE bacteria were inoculated at OD_{600}=0.001 in the indicated transport buffers and incubated at 37°C in static conditions. OD_{600} was periodically measured for up to 24 h. Transport buffers: Neutralizing Broth (NB), Neutralizing Rinse Solution (NRS), Maximum Recovery Diluent (MRD), and Amies. Data are the mean ± SD (error bars) of three independent experiments.

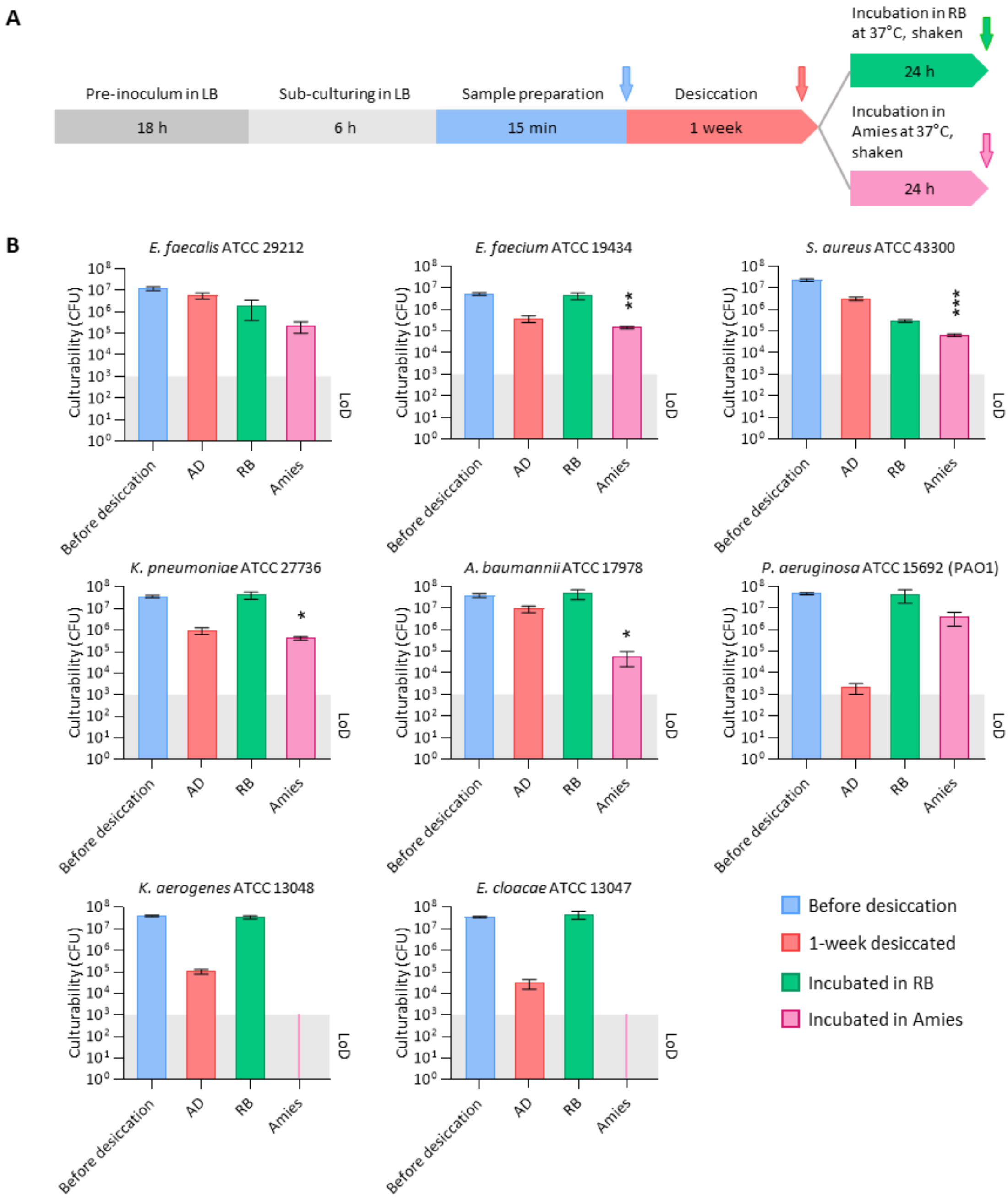

FIG S7. Resuscitation assay in RB and Amies solution. (**A**) Experimental timeline of the resuscitation assay in RB and in Amies. Bacterial strains were air-dried for one week on glass. After desiccation, cells were collected using a nylon swab soaked in RB or Amies and incubated at 37°C for 24 h with shaking (resuscitation). Colored arrows indicate the sampling times for CFU count: cyan, after sample preparation in double-distilled water; red, after one week of desiccation; green, after resuscitation in RB; pink, after resuscitation in Amies. (**B**) Cultivability of cells before desiccation (cyan), after one-week desiccation (AD, red), and after incubation at 37°C in the indicated buffers (RB, green; Amies, pink). The grey area indicates the limit of detection (LoD), corresponding to 1×10^3 CFU, with pink segments representing CFU values below the LoD after resuscitation. Data are the mean ± SD (error bars) of three independent experiments. Asterisks indicate statistically significant differences between CFU counts after resuscitation in RB and Amies. Statistically significant differences (*$P < 0.05$; **$P < 0.01$; ***$P < 0.001$; ****$P < 0.0001$) were determined using the unpaired *t*-test

Script for quantification of bacterial membrane integrity

```
// Check if a ROI exists
if (!isOpen("ROI Manager")) {
    exit("Open the ROI Manager and add a multipoint ROI.");
}

roiManager("select", 0);  // Select the multipoint ROI
getSelectionCoordinates(x, y);  // Get all the points from the ROI

n = x.length;

setResult("X", 0, "X");
setResult("Y", 0, "Y");
setResult("Red", 0, "Red");
setResult("Green", 0, "Green");

for (i = 0; i < n; i++) {
    px = getPixel(x[i], y[i]);
    red   = (px & 0xff0000) >> 16;
    green = (px & 0x00ff00) >> 8;
    blue  = (px & 0x0000ff);

    setResult("X", i+1, x[i]);
    setResult("Y", i+1, y[i]);
    setResult("Red", i+1, red);
    setResult("Green", i+1, green);
}

updateResults();
```

Protocol

Surface sampling for the detection of Gram-negative bacteria in VBNC state

Materials

Equipment and consumables

- Nylon swab (Copan) immersed in 2 mL RB
- Sampling templates (Copan), 10×10 cm (100 cm^2) and/or 5×4 cm (20 cm^2)
- Micropipette (20-200 μL) or plastic disposable graduated transfer Pasteur pipette (100 μL)
- Sterile disposable spatula
- TSA (Tryptic Soy Agar) plate
- Incubator set at 37°C with shaking
- Laminar flow hood or biosafety cabinet

Resuscitation Buffer (RB)

RB is the M9 basal salts solution (7 g/L Na_2HPO_4, 3 g/L KH_2PO_4, 0.5 g/L NaCl, 1 g/L NH_4Cl) supplemented with 0.02 g/L of $CaCl_2$ and 0.12 g/L $MgSO_4$ (Lucidi et al., 2025). Dispense 2 mL of RB to each swab-containing tube under sterile conditions (operate in a laminar flow hood or biosafety cabinet).

Method

1. **Selection of sampling area**

   Select the specified area of the surface to be examined and define the area using the sampling template (100 cm^2 or 20 cm^2, depending on the suspected level of contamination). The selected area should be representative of the sampled surface. For non-flat surfaces and surfaces not defined by a numerical size, the sampled area must be clearly described.

2. **Sampling**

2.1 Place the tube containing the swab in an upright position and ensure that the RB inside is at the bottom of the tube (if necessary, shake the tube downward to collect all the liquid at the bottom).

2.2 Press the tip of the swab against the inner wall of the tube to remove excess RB; the swab should be moist and evenly soaked with RB.

2.3 Place the sampling template (either 100 cm^2 or 20 cm^2) on the surface to be sampled.

2.4 Place the swab tip on the surface and sample the defined area (e.g., ≤100 cm^2) while rotating the swab between thumb and forefinger. For flat surfaces, the sampling should be performed

horizontally and vertically, *e.g.* 10 times in each direction. For small or hard-to-reach areas, ensure all parts, including crevices and gaps, are covered.

2.5 Return the swab to the sterile tube containing 2 mL of RB and close securely to keep it moist until analysis. Since RB contains no carbon source, samples do not require transport or storage at cold temperatures before analysis. Ensure that the transport or storage temperature is ≤ 37°C.

3. Resuscitation

Incubate the tube containing the swab immersed in RB at 37°C for 24 hours under shaking at 200 rpm.

4. Sample plating and incubation

4.1 Using a sterile pipette, transfer 100 μL of RB from point (3) onto a TSA plate and evenly spread the sample with a sterile spatula.

The volume of RB plated on the agar can be adjusted based on the operator's specific needs. For surfaces where high microbial contamination is expected, serial dilutions may be performed before plating. For surfaces where even the presence of a single bacterial cell may pose a risk, it is recommended to plate larger volumes.

Different culture media can also be used for plating according to the operator's needs. If the sampling is aimed at detecting a specific pathogen, selective media that allow isolation of the target bacterium can be used in addition to the TSA plate.

4.2 Incubate the plates at 37°C for up to 72 hours to allow colony formation.

The incubation temperature and time for the plates inoculated with RB can be adjusted depending on the bacterial species being selected.

5. Expression of results and calculation

5.1 Count the number of colonies (CFUs) on each plate.

5.2 Calculate the number of CFUs per sampled surface area (Ns) using the formula:

$$\mathbf{Ns = (N \times D) / A}$$

Where:

- **N** is the number of CFUs counted on the Petri dish
- **A** is the sampled surface area in square centimeters (*e.g.* 100 cm^2 for the 10×10 cm template and 20 cm^2 for the 5×4 cm template)
- **D** is the inverse of the dilution plated (in case 100 μL of a total volume of 2 mL is plated, 1/20 of the sample is used, so D = 20).

References

Alvear-Daza JJ, García-Barco A, Osorio-Vargas P, Gutiérrez-Zapata HM, Sanabria J, Rengifo-Herrera JA. Resistance and induction of viable but non culturable states (VBNC) during inactivation of *E. coli* and *Klebsiella pneumoniae* by addition of H_2O_2 to natural well water under simulated solar irradiation. Water Res. 2021 Jan 1;188:116499. doi: 10.1016/j.watres.2020.116499.

Bai H, Zhao F, Li M, Qin L, Yu H, Lu L, Zhang T. Citric acid can force *Staphylococcus aureus* into viable but nonculturable state and its characteristics. Int J Food Microbiol. 2019 Sep 16;305:108254. doi: 10.1016/j.ijfoodmicro.2019.108254.

Bascomb S, Lapage SP, Willcox WR, Curtis MA. Numerical classification of the tribe *Klebsielleae*. J Gen Microbiol. 1971 Jun;66(3):279-95. doi: 10.1099/00221287-66-3-279.

Bédard E, Charron D, Lalancette C, Déziel E, Prévost M. Recovery of *Pseudomonas aeruginosa* cultivability following copper- and chlorine-induced stress. FEMS Microbiol Lett. 2014 Jul;356(2):226-34. doi: 10.1111/1574-6968.12494.

Bonchi C, Frangipani E, Imperi F, Visca P. Pyoverdine and proteases affect the response of *Pseudomonas aeruginosa* to gallium in human serum. Antimicrob Agents Chemother. 2015 Sep;59(9):5641-6. doi: 10.1128/AAC.01097-15.

Bugg TD, Wright GD, Dutka-Malen S, Arthur M, Courvalin P, Walsh CT. Molecular basis for vancomycin resistance in *Enterococcus faecium* BM4147: biosynthesis of a depsipeptide peptidoglycan precursor by vancomycin resistance proteins VanH and VanA. Biochemistry. 1991 Oct 29;30(43):10408-15. doi: 10.1021/bi00107a007.

Cheng S, Su R, Song L, Bai X, Yang H, Li Z, Li Z, Zhan X, Xia X, Lü X, Shi C. Citral and trans-cinnamaldehyde, two plant-derived antimicrobial agents can induce *Staphylococcus aureus* into VBNC state with different characteristics. Food Microbiol. 2023 Jun;112:104241. doi: 10.1016/j.fm.2023.104241.

Dopp E, Richard J, Dwidjosiswojo Z, Simon A, Wingender J. Influence of the copper-induced viable but non-culturable state on the toxicity of *Pseudomonas aeruginosa* towards human bronchial epithelial cells in vitro. Int J Hyg Environ Health. 2017 Nov;220(8):1363-1369. doi: 10.1016/j.ijheh.2017.09.007.

Gin KY, Goh SG. Modeling the effect of light and salinity on viable but non-culturable (VBNC) *Enterococcus*. Water Res. 2013 Jun 15;47(10):3315-28. doi: 10.1016/j.watres.2013.03.021.

Heim S, Lleo M, Bonato B, Guzman CA, Canepari P. The viable but nonculturable state and starvation are different stress responses of *Enterococcus faecalis*, as determined by proteome analysis. J Bacteriol. 2002 Dec;184(23):6739-45. doi: 10.1128/JB.184.23.6739-6745.2002.

Hijazi S, Visaggio D, Pirolo M, Frangipani E, Bernstein L, Visca P. Antimicrobial Activity of Gallium Compounds on ESKAPE Pathogens. Front Cell Infect Microbiol. 2018 Sep 10;8:316. doi: 10.3389/fcimb.2018.00316.

Hormaeche E and Edwards PR. Proposal for the rejection of the generic name Cloaca Castellani and Chalmers, and proposal of *Enterobacter* as a generic name with designation of the type species and its type culture. Int J Syst Evol Microbiol. 1960. 10, 75-76. doi: 10.1099/0096266X-10-2-75

Jiang Q, Li H, Wan K, Ye C, Yu X. Quantification and antibiotic resistance risk assessment of chlorination-residual viable/VBNC *Escherichia coli* and *Enterococcus* in on-site hospital wastewater treatment system. Sci Total Environ. 2023 May 10;872:162139. doi: 10.1016/j.scitotenv.2023.

König P, Wilhelm A, Schaudinn C, Poehlein A, Daniel R, Widera M, Averhoff B, Müller V. The VBNC state: a fundamental survival strategy of *Acinetobacter baumannii*. mBio. 2023 Oct 31;14(5):e0213923. doi: 10.1128/mbio.02139-23.

Kvich L, Fritz B, Crone S, Kragh KN, Kolpen M, Sønderholm M, Andersson M, Koch A, Jensen PØ, Bjarnsholt T. Oxygen Restriction Generates Difficult-to-Culture *P. aeruginosa*. Front Microbiol. 2019 Aug 29;10:1992. doi: 10.3389/fmicb.2019.01992.

Li J, Liu C, Wang S, Mao X. *Staphylococcus aureus* enters viable-but-nonculturable state in response to chitooligosaccharide stress by altering metabolic pattern and transmembrane transport function. Carbohydr Polym. 2024 Apr 15;330:121772. doi: 10.1016/j.carbpol.2023.121772.

Li Y, Huang TY, Mao Y, Chen Y, Shi F, Peng R, Chen J, Yuan L, Bai C, Chen L, Wang K, Liu J. Study on the Viable but Non-culturable (VBNC) State Formation of *Staphylococcus aureus* and Its Control in Food System. Front Microbiol. 2020 Nov 26;11:599739. doi: 10.3389/fmicb.2020.599739.

Liao X, Liu D, Ding T. Nonthermal Plasma Induces the Viable-but-Nonculturable State in *Staphylococcus aureus* via Metabolic Suppression and the Oxidative Stress Response. Appl Environ Microbiol. 2020 Feb 18;86(5):e02216-19. doi: 10.1128/AEM.02216-19.

Lleo M, Bonato B, Tafi MC, Caburlotto G, Benedetti D, Canepari P. Adhesion to medical device materials and biofilm formation capability of some species of enterococci in different physiological states. FEMS Microbiol Lett. 2007 Sep;274(2):232-7. doi: 10.1111/j.1574-6968.2007.00836.x.

Lleò MM, Benedetti D, Tafi MC, Signoretto C, Canepari P. Inhibition of the resuscitation from the viable but non-culturable state in *Enterococcus faecalis*. Environ Microbiol. 2007 Sep;9(9):2313-20. doi: 10.1111/j.1462-2920.2007.01345.x.

Lleò MM, Bonato B, Benedetti D, Canepari P. Survival of enterococcal species in aquatic environments. FEMS Microbiol Ecol. 2005 Oct 1;54(2):189-96. doi: 10.1016/j.femsec.2005.03.016. PMID: 16332318.

Lleò MM, Bonato B, Signoretto C, Canepari P. Vancomycin resistance is maintained in enterococci in the viable but nonculturable state and after division is resumed. Antimicrob Agents Chemother. 2003 Mar;47(3):1154-6. doi: 10.1128/AAC.47.3.1154-1156.2003.

Lleò MM, Bonato B, Tafi MC, Signoretto C, Boaretti M, Canepari P. Resuscitation rate in different enterococcal species in the viable but non-culturable state. J Appl Microbiol. 2001 Dec;91(6):1095-102. doi: 10.1046/j.1365-2672.2001.01476.x.

Lucidi M, Capecchi G, Spagnoli C, Basile A, Artuso I, Persichetti L, Fardelli E, Capellini G, Visaggio D, Imperi F, Rampioni G, Leoni L, Visca P. The response to desiccation in *Acinetobacter baumannii*. Virulence. 2025 Dec;16(1):2490209. doi: 10.1080/21505594.2025.2490209.

Mangiaterra G, Cedraro N, Vaiasicca S, Citterio B, Frangipani E, Biavasco F, Vignaroli C. Involvement of Acquired Tobramycin Resistance in the Shift to the Viable but Non-Culturable State in *Pseudomonas aeruginosa*. Int J Mol Sci. 2023 Jul 18;24(14):11618. doi: 10.3390/ijms241411618.

Mangiaterra G, Cedraro N, Vaiasicca S, Citterio B, Galeazzi R, Laudadio E, Mobbili G, Minnelli C, Bizzaro D, Biavasco F. Role of Tobramycin in the Induction and Maintenance of Viable but Non-Culturable *Pseudomonas aeruginosa* in an In Vitro Biofilm Model. Antibiotics (Basel). 2020 Jul 10;9(7):399. doi: 10.3390/antibiotics9070399.

Pasquaroli S, Citterio B, Cesare AD, Amiri M, Manti A, Vuotto C, Biavasco F. Role of daptomycin in the induction and persistence of the viable but non-culturable state of *Staphylococcus aureus* biofilms. Pathogens. 2014 Sep 18;3(3):759-68. doi: 10.3390/pathogens3030759.

Pasquaroli S, Zandri G, Vignaroli C, Vuotto C, Donelli G, Biavasco F. Antibiotic pressure can induce the viable but non-culturable state in *Staphylococcus aureus* growing in biofilms. J Antimicrob Chemother. 2013 Aug;68(8):1812-7. doi: 10.1093/jac/dkt086.

Qi Z, Huang Z, Liu C. Metabolism differences of biofilm and planktonic *Pseudomonas aeruginosa* in viable but nonculturable state induced by chlorine stress. Sci Total Environ. 2022 May 15;821:153374. doi: 10.1016/j.scitotenv.2022.153374.

Qi Z, Liu C. Metabolic characteristics and markers in viable but nonculturable state of *Pseudomonas aeruginosa* induced by chlorine stress. Environ Res. 2022 Nov;214(Pt 3):114111. doi: 10.1016/j.envres.2022.114111.

Qi Z, Sun N, Liu C. Glyoxylate cycle maintains the metabolic homeostasis of *Pseudomonas aeruginosa* in viable but nonculturable state induced by chlorine stress. Microbiol Res. 2023 May;270:127341. doi: 10.1016/j.micres.2023.127341.

Sahm DF, Kissinger J, Gilmore MS, Murray PR, Mulder R, Solliday J, Clarke B. In vitro susceptibility studies of vancomycin-resistant *Enterococcus faecalis*. Antimicrob Agents Chemother. 1989 Sep;33(9):1588-91. doi: 10.1128/AAC.33.9.1588.

Sambrook J, Fritsch EF, Maniatis T. Molecular cloning: a laboratory manual (2nd ed.), Cold Spring Harbor Laboratory Press, Cold Spring Harbor, N.Y (1989)

Schleifer, K. H. and Kilpper-Balz, R. Transfer of *Streptococcus faecalis* and Streptococcus faecium to the genus *Enterococcus* norn. rev. as *Enterococcus faecalis* comb. nov. and *Enterococcus faecium* comb. Nov. Int. J Syst Evol Microbiol. 1984. 34, 31-34. https://doi.org/10.1099/00207713-34-1-31.

Signoretto C, Burlacchini G, Pruzzo C, Canepari P. Persistence of *Enterococcus faecalis* in aquatic environments via surface interactions with copepods. Appl Environ Microbiol. 2005 May;71(5):2756-61. doi: 10.1128/AEM.71.5.2756-2761.2005.

Smith MG, Gianoulis TA, Pukatzki S, Mekalanos JJ, Ornston LN, Gerstein M, Snyder M. New insights into *Acinetobacter baumannii* pathogenesis revealed by high-density pyrosequencing and transposon mutagenesis. Genes Dev. 2007 Mar 1;21(5):601-14. doi: 10.1101/gad.1510307.

Villa L, Feudi C, Fortini D, García-Fernández A, Carattoli A. Genomics of KPC-producing *Klebsiella pneumoniae* sequence type 512 clone highlights the role of RamR and ribosomal S10 protein mutations in conferring tigecycline resistance. Antimicrob Agents Chemother. 2014;58(3):1707-12. doi: 10.1128/AAC.01803-13.

Wang L, Ye C, Guo L, Chen C, Kong X, Chen Y, Shu L, Wang P, Yu X, Fang J. Assessment of the UV/Chlorine Process in the Disinfection of *Pseudomonas aeruginosa*: Efficiency and Mechanism. Environ Sci Technol. 2021 Jul 6;55(13):9221-9230. doi: 10.1021/acs.est.1c00645.

Wang M, Ateia M, Hatano Y, Yoshimura C. Regrowth of *Escherichia coli* in environmental waters after chlorine disinfection: shifts in viability and cultivability. Environ Sci (Camb). 2022 May 19;8(7):1521-1534. doi: 10.1039/d1ew00945a.

Yadav M, Dhyani S, Joshi P, Awasthi S, Tanwar S, Gupta V, Rathore DK, Chaudhuri S. Formic acid, an organic acid food preservative, induces viable-but-non-culturable state, and triggers new

Antimicrobial Resistance traits in *Acinetobacter baumannii* and *Klebsiella pneumoniae*. Front Microbiol. 2022 Nov 24;13:966207. doi: 10.3389/fmicb.2022.966207.

Yan H, Li M, Meng L, Zhao F. Formation of viable but nonculturable state of *Staphylococcus aureus* under frozen condition and its characteristics. Int J Food Microbiol. 2021 Nov 2;357:109381. doi: 10.1016/j.ijfoodmicro.2021.109381.

Yang D, Jiang Z, Meng Q, Wang S, Pan H, Rao L, Liao X. Analyzing the pressure resistant, sublethal injury and resuscitable viable but non-culturable state population of *Escherichia coli*, *Staphylococcus aureus, Bacillus amyloliquefaciens* and *Lactiplantibacillus plantarum* under high pressure processing. Food Res Int. 2023 Nov;173(Pt 1):113336. doi: 10.1016/j.foodres.2023.113336.

Zhang B, Fu Y, Wang F, Jin P, Xu P, Li H, Xu X, Shen C. The risk of viable but non-culturable (VBNC) enterococci and antibiotic resistance transmission during simulated municipal sludge composting. Waste Manag. 2024 Jun 30;183:1-9. doi: 10.1016/j.wasman.2024.04.048.

Zhang M, Wang X, Deng X, Zheng S, Zhang W, He JZ, Yu X, Feng M, Ye C. Viable but non-culturable state formation and resuscitation of different antibiotic-resistant *Escherichia coli* induced by UV/chlorine. Water Res. 2024 Sep 1;261:122011. doi: 10.1016/j.watres.2024.122011.7

Zhang S, Ye C, Lin H, Lv L, Yu X. UV disinfection induces a VBNC state in *Escherichia coli* and *Pseudomonas aeruginosa*. Environ Sci Technol. 2015 Feb 3;49(3):1721-8. doi: 10.1021/es505211e.

Zhao S, Dou C, Zhang J, Huang L, Gao Y, Du B, Cui X, Zhao H, Xue G, Ke Y, Gan L, Feng J, Feng Y, Cui J, Yan C, Xu Z, Fu T, Yu Z, Yang Y, Yuan J, You F. Multiple factors trigger the formation and resuscitation of the VBNC state in alcohol-producing *Klebsiella pneumoniae*. Appl Environ Microbiol. 2024 Jul 24;90(7):e0055724. doi: 10.1128/aem.00557-24.
